# Understanding the causes and consequences of low statin adherence: evidence from UK Biobank primary care data

**DOI:** 10.1186/s12916-025-04228-2

**Published:** 2025-07-22

**Authors:** Deniz Türkmen, Xiaoran Liang, Jane A. H. Masoli, Dipender Gill, Luke C. Pilling, Jack Bowden

**Affiliations:** 1 Department of Clinical and Biomedical Sciences, University of Exeter, Exeter, UK; 2 Royal Devon University Healthcare NHS Foundation Trust, Barrack Road, Exeter, EX2 5DW UK; 3 Department of Epidemiology and Biostatistics, School of Public Health, Imperial College London, 90 Wood Lane, London, W12 0BZ UK

**Keywords:** Cardiovascular, Statin, Adherence, Primary care, Pharmacogenomics, Clinical outcomes

## Abstract

**Background:**

Statins are prescribed to lower LDL cholesterol. Clinical guidelines recommend 30–50% reduction within 3 months, yet many patients do not achieve this. We investigated predictors of LDL-c reduction, treatment adherence, and adverse clinical outcomes in a sample of UK Biobank participants.

**Methods:**

We analysed 76,000 UK Biobank participants prescribed atorvastatin or simvastatin in primary care: 41,000 had LDL-c measurements before statin initiation (median = 16 days prior, IQR = 28) and within a year of starting treatment (median = 89 days, IQR = 125). Adherence was defined as the “proportion of days covered” (PDC). We estimated associations between PDC within 1 year of statin initiation, genetic factors, post-treatment LDL-c reduction, and clinical adverse outcomes. For 13,000 patients with ≥ 3 LDL-c measures, we used inverse probability of treatment weighting methods to estimate the effect of sustained adherence intervention on LDL-c reduction longitudinally.

**Results:**

LDL-c reduction following statin initiation was predicted by time until the 1st measurement (up to 26% greater reduction if returned ≤ 3 months vs > 3 months), PDC (up to 38% reduction when PDC > 95% [high] vs. 15% when PDC < 50% [low]), and the pharmacogenetic variant SLCO1B1*5 (lowest reduction in CC-allele: 37% versus TT-allele: 39.5%). Longitudinal causal modelling showed that the most recent PDC measure exerted the largest influence on overall LDL-c reduction, followed by the initial PDC.

Genetic predictors of reduced PDC included liability to schizophrenia (Coef_top 20%_ − 1.94, 95%CI − 2.69 to − 1.19), while genetic liability to cardiovascular diseases increased PDC (Coef_top 20%_1.30, 95%CI 0.55 to 2.05). High PDC was associated with increased risk of incident iron deficiency anaemia (HR 1.30, 95%CI 1.09–1.54) and cataract (HR 1.20, 95%CI 1.07–1.34), and decreased risk of incident coronary heart disease (HR 0.78, 95%CI 0.73–0.84).

**Conclusions:**

We identify substantial variability in the time to first on-treatment LDL-c measurements and also in adherence to statin medication, highlighting a gap between NHS guidelines, LDL-c monitoring, and statin adherence. We show its subsequent impact on long-term health, demonstrating the potential effect of targeted interventions to improve adherence. We identify important predictors of reduced statin effectiveness, including pharmacogenetic variants, polygenic scores, but most of all, adherence. Tailored statin therapy strategies with patient education on statin indication and adherence could optimize treatment efficacy, safety, and long-term clinical outcomes.

**Supplementary Information:**

The online version contains supplementary material available at 10.1186/s12916-025-04228-2.

## Background

Cardiovascular diseases (CVDs) remain the leading cause of mortality and morbidity in adults globally [1]. Low-density lipoprotein cholesterol (LDL-c), a key modifiable risk factor, can be lowered with statins, reducing CVD risk by ~ 22% per 1 mmol/L (18 mg/dL) reduction [2]. National guidelines, including the UK National Institute for Health and Care Excellence (NICE) and the American Heart Association (AHA), recommend a 30–50% target LDL-c reduction for those aged 40–75 with a high CVD risk with a follow-up lipid check at 2–3 months post-statin initiation [3–5]. In the UK, for secondary prevention, the target LDL-c is < 2 mmol/L (36 mg/dL). Many individuals with detected hypercholesterolaemia are not followed up in the desired time frame and do not achieve these targets [6–9]. Adherence to statin therapy in real-world studies can be < 50% within the first year of treatment [10–12], far lower than in randomized controlled trials (RCT) conducted with close clinical monitoring and mostly concentrating on patients after an CVD event or hospitalization [13, 14]. Poor long-term adherence to statin therapy is associated with higher hospitalization rates [15], yet the patient-related causes and consequences of statin adherence in routine primary care remains unclear.

Recent trials [16, 17] show those at high genetic risk for CVD experience greater benefits from statins in primary prevention, but whether this effect is pharmacogenetic or simply an artefact of increased pre-treatment LDL-c levels is unclear. Understanding any effect of pharmacogenetic variants and polygenic scores (aggregated over multiple genetic loci) on statin adherence and LDL-c reduction is of vital importance, since they offer the potential for stratified medicine to better support the identification and optimization of treatment outcomes in high-risk populations. Pharmacogenetic variants in *SLCO1B1* and other genes that impact risk of muscle symptoms [18] or discontinuation [19] may also therefore affect adherence. Recent largescale studies have investigated socio-demographic and genetic risk factors of statin adherence [20], or genome-wide association studies of lipid response to statins [21], but have not considered adherence, LDL-c reduction, and adverse clinical outcomes in parallel. To address this, we conducted an extensive analysis of 76,000 UK Biobank participants prescribed statin medication in the linked primary care records. We estimated the impact of time to first on-treatment follow-up, pharmacogenetic variants, polygenic scores, and adherence on LDL-c reduction within 1 year of initiating statin treatment. We also estimated the impact of low adherence on adverse clinical outcomes. To further evaluate the effect of interventions to increase statin adherence on LDL-c levels over time, we performed longitudinal causal modelling of individual patient LDL-c trajectories, paired with inverse probability of treatment weighting (IPTW) methods [22] to control for observable time-varying confounders of adherence and LDL-c.

## Methods

### Cohort: UK Biobank

**Fig. 1:**
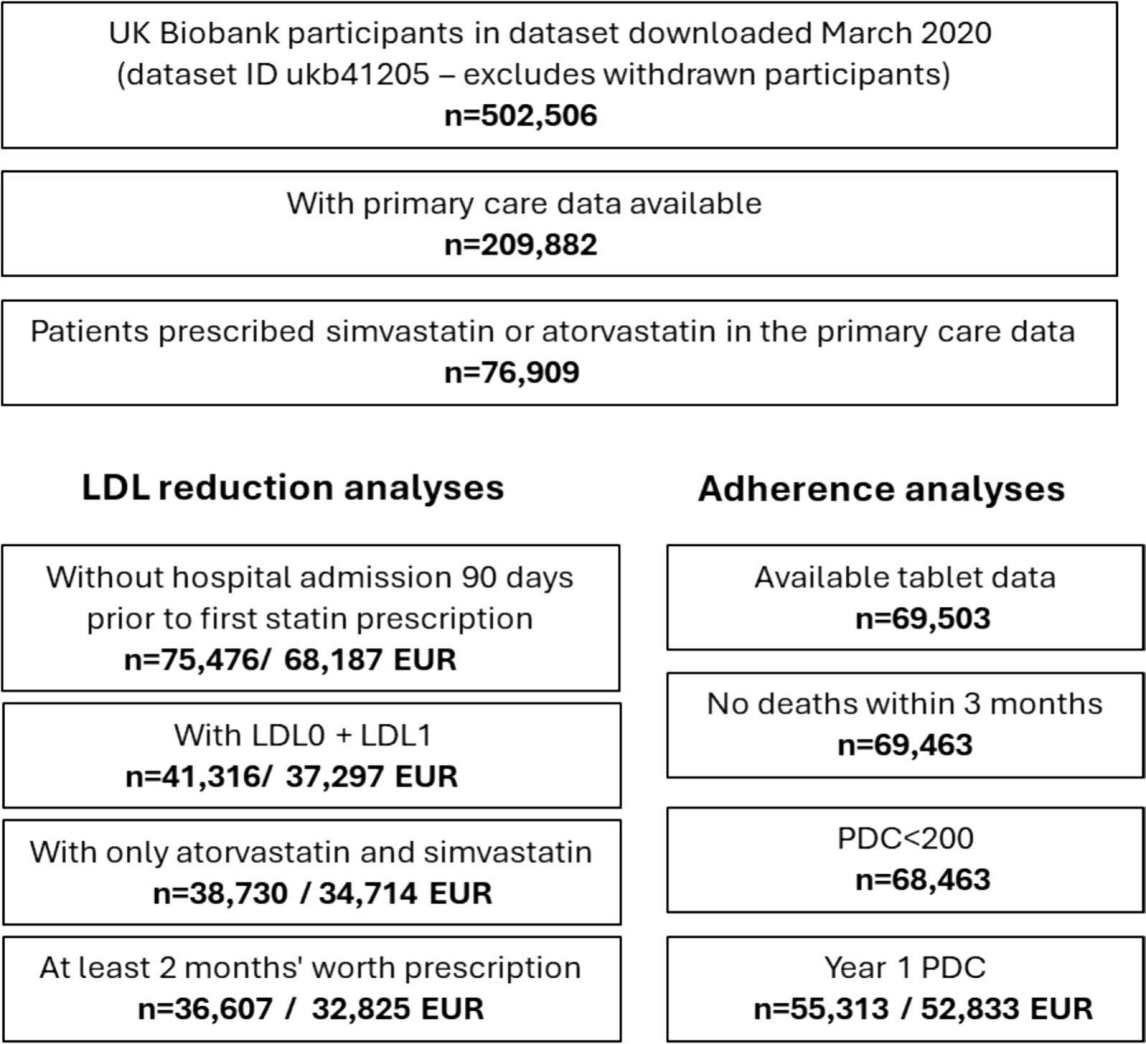
Flowchart of the UK Biobank cohort. LDL0: pre-statin LDL-c measurement, LDL1: post-statin LDL-c measurement, EUR: Genetically European ancestry. We primarily studied two questions: 1) What predicts adherence? (e.g. time to first LDL-c measurement, polygenic scores, pharmacogenetic variant SLCO1B1*5). 2) How does adherence impact outcomes (e.g. LDL-c, clinical outcomes)? Of 76,909 patients, 69,503 had the information about their prescriptions’ quantity (e.g. number of tablets). We excluded patients who died within 3 months of starting statins and those with a >200% as values exceeding 200% likely indicate data errors or irregular medication supply patterns

The UK Biobank (UKB) recruited 503,325 community-based volunteers aged 40–70 from Wales, Scotland, or England over the period 2006–2010. Participants provided blood samples for genetic and biochemical analyses. This study involves two analyses: [1] using the linked primary care data from General Practice (GP) available in 230,096 participants (45.7% of UKB) (Fig. 1) to examine LDL-c reduction and [2] incorporating secondary care data (hospital) with the GP data for the incident outcomes.

GP data includes drug name, date of prescription, number of tablets, and drug code (in Clinical Read version 2, British National Formulary (BNF) or Dictionary of Medicines and Devices (DM + D) format, depending on provider) and are up to September 2017 (EMIS/Vision system in Wales) and August 2016 (TPP system supplier in England). We included simvastatin and atorvastatin prescriptions, classifying first doses by NICE guidelines [3]: 8% low-intensity, 76% medium-intensity, and 16% high-intensity in the UKB cohort (Additional file 1: Tables 1 and 2).

We identified primary and secondary care-diagnosed CVDs (Abdominal Aortic Aneurysm, Coronary Heart Disease, Ischemic Stroke, Ischemic Stroke or Transient Ischemic Attack, Peripheral Arterial Disease, All Types of Stroke, and Transient Ischemic Attack) based on clinical guidelines for initiating statin treatment for secondary prevention [23]. Additional file 2: Table 1 for the ICD-10 codes.

### Genetic data

UKB obtained 805,426 directly genotyped variants via the Affymetrix Axiom UKB array (in 438,427 participants) and the Affymetrix UKBiLEVE array (in 49,950 participants). Imputation was performed by the central UKB team for 487,442 participants and ~ 96 million genetic variants were obtained. Neither participants’ nor their healthcare providers received genotype data as part of the study, so this information could not directly influence treatment. UKB is predominantly of European genetic ancestry, with 451,367 (93% of the 487,000 with genetic data) participants genetically similar to the 1000Genomes EUR reference population (“European-like”). We restricted our analysis to this sample as including genetically diverse individuals might introduce bias due to population stratification (this was identified via Principal Components Analysis using the 1000Genomes reference panels, see [24] for details).

We also included polygenic scores (PGS) derived by Genomics PLC [25] (UKB category 301): 8 PGS for relevant biomarkers and patient characteristics (e.g. LDL-c, BMI) and 28 PGS for genetic liability to disease (e.g. CVD, schizophrenia). Traits calculated with standard method (scores calculated for all participants in UKB, trained on only external data) in European populations were included. See Additional file 2: Table 2 for the details.

Adherence as measured by proportion of days covered (PDC). For each patient with a history of statin treatment initiation as well as pre- and post-treatment LDL-c measures (*n* = 69,503), we sought all available prescribing data. We then calculated the number of tablets prescribed to each individual in a given time period, to create the variable:

$$PDC=\frac{\mathrm{Number}\;\mathrm{of}\;\mathrm{tablets}\;\mathrm{prescribed}\;\mathrm{in}\;\mathrm{study}\;\mathrm{period}}{\mathrm{Length}\;\mathrm{of}\;\mathrm{study}\;\mathrm{period}\;\left(\mathrm{days}\right)}\times100$$

We first focused on adherence in the first year of treatment, which we call ${PDC}_{1}$. If patients received a new prescription that extended past the first 12 months, we included the time until the next prescription in the “length” calculation for ${PDC}_{1}$. However, if there were no subsequent prescriptions, we excluded that prescription from the calculation (*n* = 7446). We also excluded patients who died within 3 months of starting statins (*n* = 39) and those with a ${PDC}_{1}$ >200% (*n* = 714). See Fig. 1 for a flowchart of this process and the final number of included participants.

We used a three-category definition for ${PDC}_{1}$ when treating it as the exposure: < 50%, 50–95%, > 95% based on observed adherence patterns in the cohort and partly in line with previous research (e.g. for < 50%) [12]. In addition, when treating it as the outcome variable and estimating its predictors via multivariable regression modelling, we used the original (continuous) ${PDC}_{1}$ measure. When performing longitudinal causal modelling of individuals’ LDL-c trajectories over three post-treatment follow-up visits, continuous measures for PDC adherence over participant-specific study windows were used as covariates and in the derivation of inverse probability of treatment weights.

## Outcomes

### LDL-c change in 1 year

We obtained GP-recorded LDL-c measurements for patients on statins (see Additional file 2: Table 3 for the read codes used). We identified the baseline LDL-c measure (within 180 days and preceding first prescription: selecting the value closest to that date, to capture a baseline lipid level that reasonably reflects the patient’s pre-treatment status) and the first follow-up measure following statin initiation, restricting analyses to participants with a follow-up measure within 12 months of statin initiation. LDL-c measurements for patients prescribed their first statin within 90 days of hospital discharge for a cardiovascular event were excluded, as these prescriptions may not have been recorded by the GP. Extreme measurements (LDL-c < 1 mmol/L: *n*_LDL0_ = 1402, *n*_LDL1_ = 2081 and LDL-c > 8 mmol/L: *n*_LDL0_ = 29, *n*_LDL1_ = 13) were excluded (see Fig. 1). We defined an LDL-c change measure as the post-statin/pre-statin LDL-c ratio to directly model the outcome on the scale for which NHS guidelines are expressed:

$$LDL\;change\;\left(Y\right)=\frac{{LDL}1\;\left(\mathrm{follow}-\mathrm{up}\;\mathrm{LDL}\;\mathrm{measurement}\right)}{LDL0\;\left(\mathrm{baseline}\;\mathrm{LDL}\;\mathrm{measurement}\right)}$$

### Long-term clinical outcomes

We investigated 84 long-term clinical outcomes to identify both intended and unintended adverse events. These included all common chronic diseases lasting over 3 months with a prevalence greater than 0.5% in older adults. The specific diagnostic codes used to define these conditions are detailed in our previous study [26].

### Analyses

**Fig. 2:**
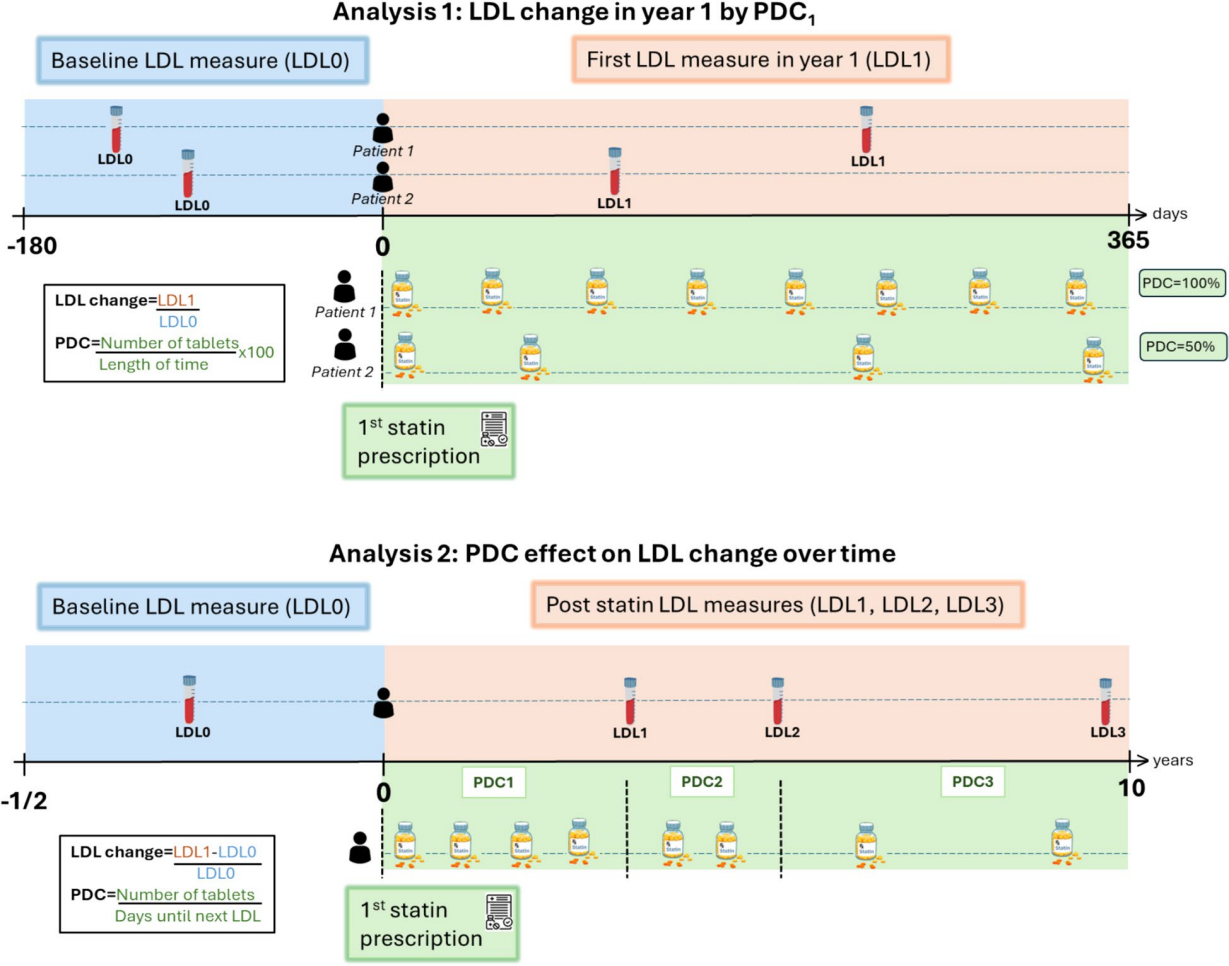
Design of the analyses. For each patient with a history of statin treatment initiation as well as pre and post treatment LDL-c measures (*n* = 69,503), we sought all available data on the date and quantity their prescription. We then calculated the number of tablets prescribed to each individual in a given time period, to create the PDC variable. Analysis 1: We estimated LDL-c change for varying the adherence in the first year of treatment, which we call (PDC_1_). We calculated PDC over the full year from treatment initiation to provide an average measure of medication adherence, as it accounts for adherence behaviour across time (e.g., pill stocking, refills etc). If patients received a new prescription just before the end of year 1, we included the time until the next prescription in the 'length' calculation for (PDC_1_). However, if there were no subsequent prescriptions, we excluded that latest prescription from the calculation (*n* = 7,446). We also excluded patients who died within 3 months of starting statins due to pre-existing illness (*n* = 39) and those with a PDC_1_ >200% (*n* = 714). Analysis 2: We attempted to evaluate how intervening to increase statin adherence would affect a person’s LDL-c change over a longer sustained period, adjusting for fixed confounders of PDC and LDL-c, as well as time-varying confounding of previous PDC and LDL-c measures on their future values. For patients prescribed statins, we included individuals with at least three post-statin LDL-c measures over a ten-year window for the analysis (*n* = 13,315)

Figure 2 provides a detailed visualization of our analysis strategy. All models in Analysis 1 are adjusted for sex, age at first statin prescription, the baseline assessment centre, first dose and education unless stated otherwise.

### Analysis 1: cross-sectional analysis of LDL-c change

#### LDL-c change explained by week of follow-up

We tested associations between LDL-c change and the time interval between their pre- and post-treatment measurements, using a linear regression model, adjusted additionally for dose of first atorvastatin and simvastatin prescribed in year 1 and the prevalent cardiovascular disease diagnoses (any CVD, binary: Abdominal Aortic Aneurysm, Coronary Heart Disease, Ischemic Stroke, Ischemic Stroke or Transient Ischemic Attack, Peripheral Arterial Disease, All Types of Stroke, and Transient Ischemic Attack). Doses were organized into a categorical variable based on NICE guidelines [3] (Additional file 1: Table 1). We repeated this analysis in the ≤ 95% and > 95% ${PDC}_{1}$ groups separately.

#### Modelling LDL-c change by genotype, PDC and time of follow-up measure

We next tested associations between LDL-c change and genetic variant rs4149056 using a linear model that allowed for a quadratic effect of time-to-follow-up on treatment in European-like participants and an interaction between time on treatment and rs4149056. We also conducted linear regression analyses to test the associations between polygenic scores and LDL-c change. We additionally adjusted for 10 genetic principal components (PCs) in these two analyses.

Next, we tested the association between LDL-c change and ${PDC}_{1}$ using the same model, but with PDC in place of rs4149056. This analysis did not adjust for genetic PCs but accounted for the prevalence of CVD before statin use, including abdominal aortic aneurysm, atrial fibrillation, coronary heart disease, peripheral arterial disease, all types of stroke, and thromboembolic disease. For further technical details on the statistical modelling see the Additional file 1.

### Secondary analysis

In addition to the pharmacogenetic SLCO1B1*5 variant, we also analysed previously reported [27] lipid-response variants in *SORT1 (rs646776)*,* SLCO1B1 (rs2900478: r*^2^ = 0.96 with SLCO1B1*5*)*,* LPA (rs10455872)*, and* APOE (rs445925)* as a secondary analysis to explore broader genetic influences on LDL-c response beyond the primary pharmacogenetic variant SLCO1B1*5 and pharmacokinetic pathways.

### Analysis 2: longitudinal analysis on LDL-c trajectories

#### Causal modelling of hypothetical LDL-c change via sustained PDC intervention

We applied and refined causal modelling approaches to evaluate how intervening to increase statin adherence would affect a person’s LDL-c reduction over a longer sustained period, adjusting for fixed confounders of PDC and LDL-c, as well as time-varying confounding of previous PDC and LDL-c measures on their future values. For patients prescribed statins, we included individuals with at least three post-statin LDL-c measures over a 10-year window for the analysis (*n* = 13,315). We defined LDL-c reduction (LR) as the percentage change between each of their post-statin LDL-c measures and the pre-statin baseline level (denoted by ${LDL}_{0}$) as.

$$LR_k=\frac{LDL_k-LDL_0}{LDL_0}\mathrm{for}\;\mathrm{time}\;\mathrm{point}\;k\;=1,2,3$$

**Fig. 3:**
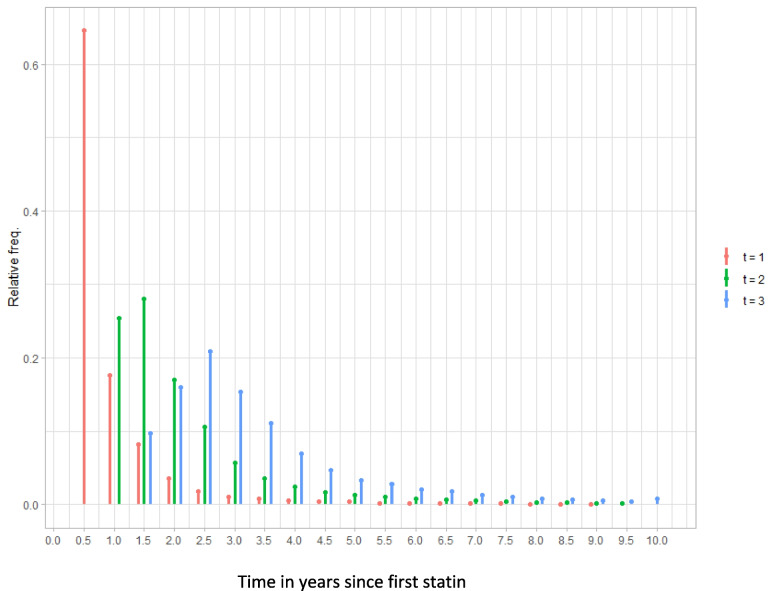
Frequency distribution of first three post-statin LDL-c measures times. The x-axis shows the time length by year, and y-axis shows the relative frequency

**Fig. 4:**
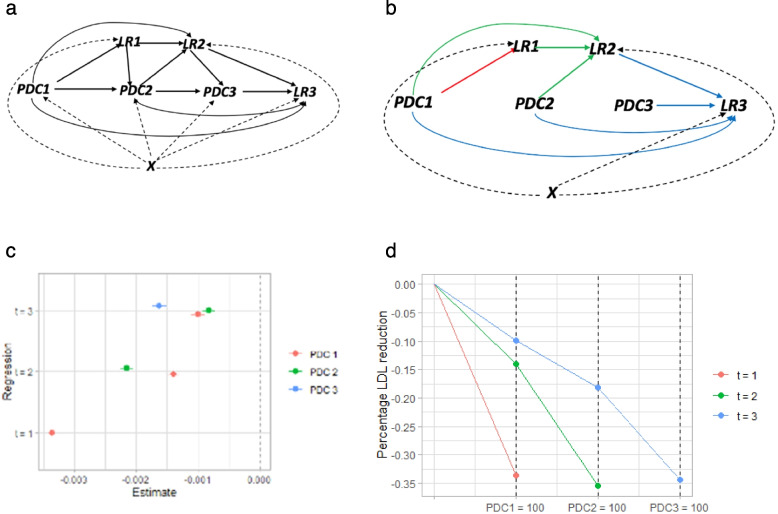
Causal diagrams illustrating the model setup of and results of the inverse probability of treatment weighting analysis.** a** Causal diagram illustrating the conditional dependencies and independencies between three adherence measurements PDC1-PDC3 and three outcome measurement LR1-LR3 in the presence of a set of confounders X. **b** Causal diagram illustrating the conditional dependencies and independencies between PDC and LR after inverse probability of treatment weighting. **c** Estimates and 95% confidence intervals of the effects of PDCs on LR for each time point from the weighted OLS using the CBPS weights. **d** The difference in percentage LDL-c reduction between the cases with all PDCs equal to 0 and all PDCs equal to 100. The red line represents the effect of PDC1 on LR1, the two segments of the green line (from left to right) represent the effect of PDC1 and PDC2 on LR2 respectively, and the three segments of the blue line (from left to right) represent the effect of PDC1, PDC2 and PDC3 on LR3 respectively

Figure 3 shows the distribution of patients’ three follow-up times over the full study window. The average follow-up time to the third LDL-c measure was 3 years and the maximum was ten. For this analysis, we redefined ${PDC}_{k}$ as the average PDC value between time point *k − 1* and *k*. For example, if *k* = 1, then *k* − 1 indexes the baseline LDL-c and *k* indexes the first follow-up LDL-c. We assumed that ${PDC}_{1}$ up to ${PDC}_{k}$ exerted additive linear effects on ${LR}_{k}$. Our goal was to estimate these effects, averaged over the study population, by adjusting for fixed and time-varying confounding. The causal diagram in Fig. 4 panel a shows the assumed data structure for our analysis.

We made the critical assumption that all the confounders of PDC and LR were observable and could be controlled for in the analysis. We included age at the first statin prescription, sex, pre-statin baseline LDL-c (i.e. ${LDL}_{0}$), education and assessment centre as fixed confounding factors. Previous PDC and LR were assumed to act as time varying confounders of future PDC and LR measures. We also included patient visit time for each post-statin LDL-c measure as the time-varying confounder. In this setting, traditional regression approaches might suffer from the so-called “post-treatment bias” when there exist time-dependent confounders that are also affected by past exposure history [28, 29]. To address this issue, we employed the inverse probability of treatment weighting (IPTW) method, which can mitigate this bias and has the additional advantage of not requiring the modelling of the potentially complex outcome–covariate relationship [28]. The IPTW method is a two-step procedure.

In stage 1, we create a pseudo-population by assigning everyone in the sample a weight so that, in this weighted pseudo-population, the exposure PDC is balanced across all the confounding covariates (see Fig. 4b for illustration). We applied the Covariate Balancing Propensity Score (CBPS) method, which provides improved robustness to model misspecification of the exposure [30] to estimate the weight for each individual. Both the parametric and non-parametric (npCBPS) versions of the method were utilized.

After obtaining the weights, we evaluated the covariate balance in the pseudo-population by calculating the weighted Pearson and Spearman correlation between each exposure and confounding covariate. Following the practice in [31], if the size of the correlation fell below the cut-off value 0.1, then the corresponding covariate was deemed sufficiently balanced. In stage 2, for each time point *k*, we estimated the effect of each ${PDC}_{1}$ to ${PDC}_{k}$ on ${LR}_{k}$ by the weighted OLS regression (regressing ${LR}_{k}$ on ${PDC}_{1}$ to ${PDC}_{k}$ simultaneously) using the weights obtained from the first step. The effect of the contemporaneous ${PDC}_{k}$ on ${LR}_{k}$ is the direct effect, while the effect of intervening on a previous PDC on ${LR}_{k}$ incorporates both the direct effect and the indirect effect via the earlier ${LR}$ measures. If a covariate failed the balancing test, we included it as a covariate in the regression [32]. See the Additional file 1 for further technical details on the causal modelling and estimation methods.

We conducted LDL-c analyses (Analysis 1 and Analysis 2) separately for patient groups with and without prevalent CVD.

### Genome wide association studies (GWAS)

We performed GWAS analysis of PDC traits using REGENIE v3.3 [33] adjusted for age, sex, assessment centres, and genotyping microarray (2 categories) to identify genetic variants predicting PDC. REGENIE adjusts for sample relatedness and population structure during analysis. We studied 16 million genetic variants from the imputed data released by UKB (category 100,319, [34]), where the imputation quality was > 30% and minor allele frequencies (MAF) were > 0.1%. *P*-values < 5*10^−8^ were taken as genome-wide significant.

### Time-to-event analysis of long-term clinical outcomes

We conducted a time-to-event analysis beginning 3 months post-statin initiation. Patients exited the model at the event date or censoring date of their hospital data. The model adjusted for sex, age at first statin prescription, education level, assessment centre, and baseline binary event diagnosis. Adherence levels were categorized as ≤ 50%, 50–95%, and > 95% and used as exposure.

We applied the Benjamini–Hochberg procedure to control the false discovery rate across all multiple tests involving different exposures and outcomes (R: p.adjust(pval, method ="BH")), and report adjusted *p*-values as “p_BH”. As iron deficiency anaemia (IDA) is more prevalent in women [35], we conducted a gender-stratified analysis for IDA.

Unless otherwise stated all analyses were performed using R v4.3.1 and the package “survival” (v3.5–8) was used for time-to-event analysis. Package “*CBPS*”* (version: 0.23)* was used for longitudinal causal analyses.

## Results

### LDL-c

Of the 76,909 UKB participants ever prescribed statins in their available primary care data (33% of all participants with primary care data), 41,316 had LDL-c measurements prior to statin initiation (< 180 days pre-statin; Mean: 32, SD: 38, Median: 16) and within 1 year of first statin prescription (post-statin LDL-c measurement; Mean: 123 days, SD: 96, Median: 92). Of these, 37,297 were of European-like genetic ancestry (Fig. 1). Mean age at statin initiation was 61 years (SD 7.3, range 40–79), and 44.4% were female (Table 1).

**Table 1:** Descriptive table of patients who have GP prescribed statin

Female (*N*, %)	32,545 (42.3%)
Treatment duration (years)	0.01–25 (mean 6.3)
Number of participants with PDC_1_	55,313
PDC_1_ < 50 (%)	8%
PDC_1_ = 50–95 (%)	33%
PDC_1_ > 95 (%)	59%
Age at first statin (mean)	61 (SD 7.3)
PDC_1_ < 50	59 (SD 7.6)
PDC_1_ = 50–95	60 (SD 7.6)
PDC_1_ > 95	62 (SD 7.1)
BMI kg/m^2^ (mean)	29 (SD 5–IQR 26–31)
PDC_1_ < 50	29 (SD 5–IQR 26–31)
PDC_1_ = 50–95	29 (SD 5–IQR 26–31)
PDC_1_ > 95	29 (SD 5–IQR 26–31)
LDL-c pre statin (GP) mmol/L (mean)	3.8 (SD 1.05)
PDC_1_ < 50	3.9 (SD 1.01)
PDC_1_ = 50–95	3.8 (SD 1.06)
PDC_1_ > 95	3.8 (SD 1.07)
No Prevalent CVD (primary prevention)	4 (SD 1)
Prevalent CVD (secondary prevention)	3.4 (SD 1)
LDL-c post statin (GP) mmol/L (mean)	2.5 (SD 0.95)
PDC_1_ < 50	3.2 (SD 1.1)
PDC_1_ = 50–95	2.6 (SD 0.9)
PDC_1_ > 95	2.3 (SD 0.8)
No Prevalent CVD (primary prevention)	2.6 (SD 0.9)
Prevalent CVD (secondary prevention)	2.3 (SD 0.8)
CVD*	
Pre statin (N)	17,849
PDC_1_ < 50 (%)	34%
PDC_1_ = 50–95 (%)	37%
PDC_1_ > 95 (%)	40%
Post statin (N)	16,250
PDC_1_ < 50 (%)	25%
PDC_1_ = 50–95 (%)	22%
PDC_1_ > 95 (%)	21%

*PDC*_*1*_, Proportion of days covered in year 1 of statin initiation, *CVD,* Cardiovascular diseases included: Abdominal Aortic Aneurysm, Coronary Heart Disease, Peripheral Arterial Disease, All Types of Strokes, and Transient Ischemic Attack. Post statin: Within 1 year of last statin prescription

### Analysis 1: cross-sectional analysis of LDL-c change

The mean pre- and post-statin LDL-c was 3.8 mmol/L (SD 1.05) and 2.5 mmol/L (SD 0.95) respectively. Individuals with pre-statin CVD (secondary prevention group) had, on average, baseline LDL-c levels 0.59 units lower (95% CI 0.62 to 0.56, *p* = 7.6 × 10 − 355) and pre-statin CVD was associated with a 0.12 unit increase in dose intensity (95% CI 0.1 to 0.13, *p* = 3.7 × 10 − 71) than those without pre-statin CVD.

Across all individuals, we found a U-shaped relationship between LDL-c reduction and the time to first follow-up, with a maximum reduction for those returning for follow-up between weeks 5-8 of 26% (95%CI 23-29) (Additional file 1: Fig. S1). The mean value was 94.7%, median is 97% (IQ 85.5-105) and the prevalence of PDCs is as follows: PDC = 50% (8%), PDC = 50-95% (33%), and PDC > 95% (59%). Stratifying the analysis by Individuals who had <=95% and >95 revealed the U-shape was driven by the former patient group rather than the latter (Additional file 1: Fig. S2).

**Fig. 5:**
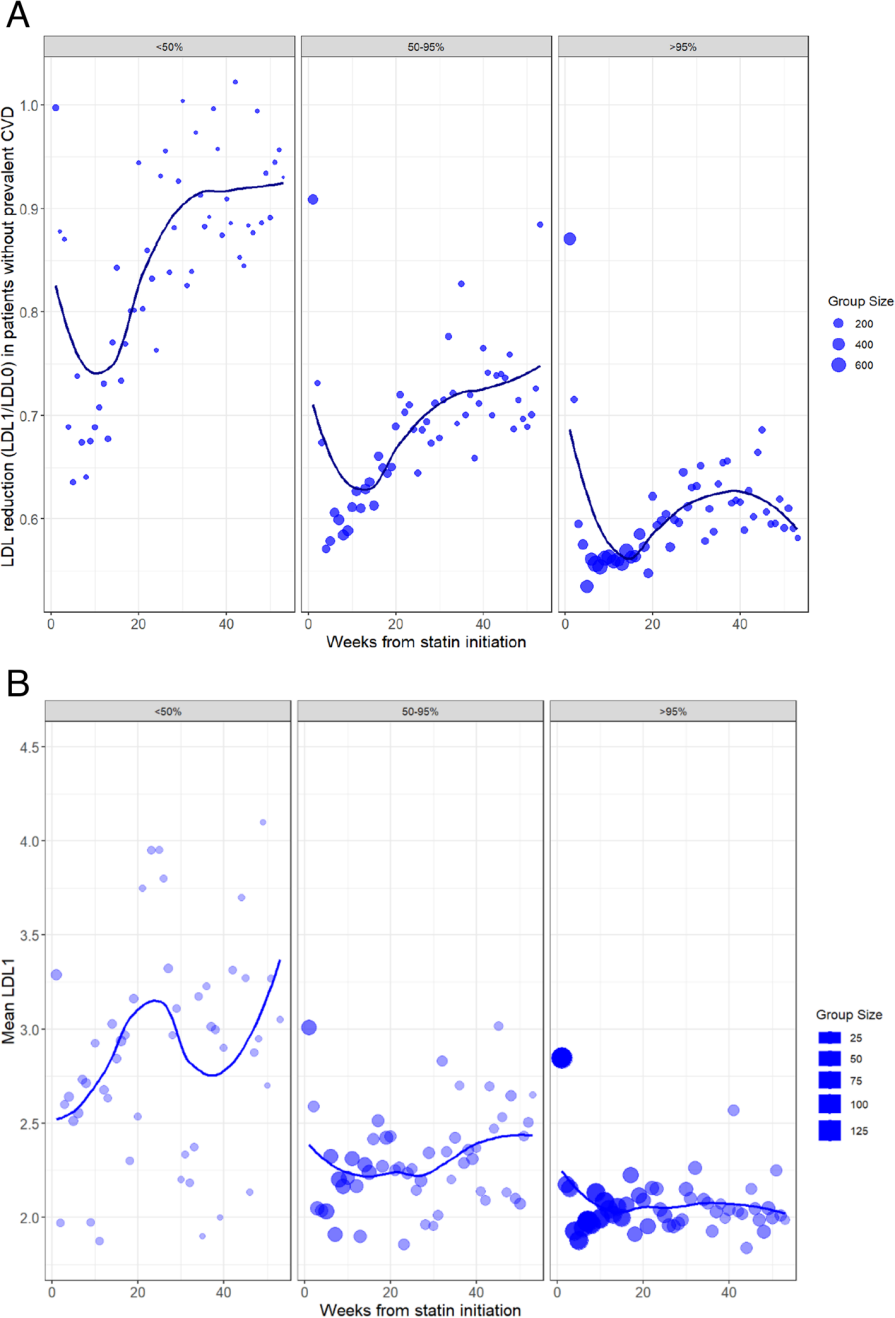
LDL-c Change in Primary Prevention & Mean LDL-c in Secondary Prevention Groups. **A** LDL-c Change in the Primary Prevention Group and Distribution of Follow-Up LDL-c Measurements by PDC Groups Change in LDL-c levels (LDL1/LDL0) after statin initiation, stratified by treatment group. Points represent the average LDL-c change for each time bin (weeks from statin initiation) in patients without prevalent cardiovascular disease. The size of the points reflects the number of patients in each group. The plot is divided by three treatment groups, categorised by the percentage of patients in each group (<50%, 50–95%, and >95% of the population). **B** Mean Post-Statin LDL-c in the Secondary Prevention Group and Distribution of Follow-Up LDL-c Measurements by PDC Groups The mean first post-statin LDL-c measurements in secondary prevention group

In the primary prevention group, the high PDC group, which was also more adherent to post-statin LDL-c follow-up (Fig. 5A), achieved the greatest LDL-c reduction, with a 40% reduction (LDL-c ratio ~ 0.6) by 15 weeks. In the secondary prevention group, post-statin LDL-c levels were reduced to below 2 mmol/L, as recommended by NICE guidelines, in 31% of individuals. Those in the high PDC group (> 95%) showed the greatest LDL-c reduction by 15 weeks, with 57% achieving LDL-c ≤ 2, compared to 23% in the low PDC group (*p* = 2 × 10⁻^31^). The group sizes and densities indicate greater adherence to LDL-c check-ups in the high PDC group, which was associated with the most significant LDL-c reduction over time (Fig. 5B).

Finally, SLCO1B1*5 genotype was seen to alter the relationship between follow-up time and LDL-c reduction: the homozygotes (i.e. rs4149046 CC genotype) had the smallest LDL-c reduction in the quadratic model in year 1 (Additional file 1: Fig. S3). See Additional file 1: Fig. S4 for the other genetic variants. The polygenic score for Alzheimer’s disease was associated with a reduced LDL-c reduction but not significant after multiple correction testing (Coef: 4.5 × 10⁻^3^, 95% CI 1.7 × 10⁻^3^ to 7.3 × 10⁻^3^, *p* = 0.002, p_bh = 0.07). Other reported variants associated with lipid reduction following statin initiation were investigated: rs10455872 in the LPA gene had the strongest associated with LDL-c change in the first year in our analysis. Additional file 2: Table 4 for the other polygenic scores.

### Analysis 2: longitudinal analysis on LDL-c trajectories

Using the weights obtained from the stage 1 weighting procedure, we examined the weighted pair-wise correlations between ${PDC}_{k}$ and each of its constituent variables with both the Pearson and Spearman correlation. All covariates except for the patient visit times had average correlations (both Pearson and Spearman) with ${PDC}_{k}$ that fell below the 0.1 threshold (Additional file 2: Tables 5 and 6). We therefore explicitly included patient visit times as covariates in the stage two weighted regressions.

Model-derived estimates of the effects of PDCs on LR with the 95% confidence intervals using the CBPS weights are shown in Fig. 4c. All three PDC measures have significant (*p* < 0.05) negative effects on LDL-c reduction (Additional file 2: Table 7). Firstly, for all three time points, the most recent PDC measure,${PDC}_{k}$, had the largest estimated effect on ${LR}_{k}$. Secondly, the initial adherence level ${PDC}_{1}$ had a larger estimated impact on ${LR}_{3}$ than${PDC}_{2}$. Thirdly, while PDCs at different time points had different estimated effects, the total effect estimate (expressed as a summation across the time points) remained remarkably constant. For illustration, we calculated the average difference in LDL-c reduction if all individuals’ PDCs were set to 100 versus if all PDCs were set to 0 in Fig. 4d. This revealed that LDL-c percentage reductions of approximately 35% can be achieved for sustained, full adherence to statins. Another relevant measure for each patient is the LDL-c reduction if they could maintain their own personal maximum PDC level across each time point compared to their observed PDC level at each time point. Our model suggests this difference would be approximately 6%. We conducted the analysis using the non-parametric option of the CBPS method (npCBPS) as well, with consistent results (Additional file 2: Table 8).

The longitudinal analysis of LDL-c changes with sustained PDC intervention in the primary prevention subgroup (*n* = 10,971) yielded results consistent with those in the main analysis of the full sample (Additional file 2: Tables 9 and 10). There was insufficient statistical power for analysis of the secondary prevention subgroup due to small sample size (*n* = 2344).

### Predictors of PDC_1_

Male participants (Coef − 0.64, 95% CI − 1.25 to − 0.04, *p* = 0.03) and current smokers (Coef − 3.66, 95% CI − 4.61 to − 2.40, *p* = 7 × 10^−14^) were less adherent (lower PDC). Participants with higher pre-statin LDL-c, higher educational attainment, more prevalent diseases, and who were older at first stain prescription were more adherent (higher PDC). BMI was not associated with PDC (Coef 0.01, 95% CI − 0.05 to 0.07). Genetic predictors of reduced PDC included liability to schizophrenia (Coef_top 20%_ − 1.94, 95%CI − 2.69 to − 1.19), c PDC (Coef_top 20%_ 1.30, 95%CI 0.55 to 2.05) (Additional file 2: Table 11). rs4149046 CC (SLCO1B1*5) was not significantly associated with PDC in linear regression models (Coef − 0.002, 95% CI − 1.56 to 1.55, *p* = 0.1). See Table 2 for the details.

**Table 2:** Associations between risk factors and PDC (proportion of days covered) as adherence measure using linear regression in GP-prescribed statin patients in UK Biobank

Exposure	*n*	Coef	95%	CI	*p*
***Observational^***					
Sex (male)		− 0.64	− 1.25	− 0.04	3.7E − 02
Age at first statin		0.34	0.30	0.39	5.8E − 54
BMI		0.01	− 0.05	0.07	7.5E − 01
LDL-c pre statin (GP)		0.55	0.30	0.80	1.3E − 05
**Smoking**					
Never smoked	24,932	*ref*	-	-	-
Ex-smoker	22,982	0.63	− 0.003	1.26	5.1E − 02
Current smoker	7014	− 3.66	− 4.61	− 2.70	7.2E − 14
**Education**					
None	15,583	*ref*	-	-	-
CSEs	1584	0.17	− 1.63	1.98	8.5E − 01
GCSEs/O-levels	6729	1.38	0.38	2.38	6.9E − 03
A-levels/NVQ/HND/HNC	9403	1.24	0.32	2.15	7.9E − 03
Prof.qual	7952	1.92	0.98	2.87	6.5E − 05
Degree	13,110	2.52	1.69	3.34	2.4E − 09
Multimorbidity*					
No add. Disease	3127	*ref*	-	-	-
1 Disease	7644	2.85	1.33	4.37	2.4E − 04
2 Diseases	9128	3.97	2.49	5.45	1.5E − 07
3 Diseases	8584	3.87	2.38	5.36	3.7E − 07
4 Diseases	7087	4.30	2.78	5.82	3.2E − 08
4 + Diseases	19,743	4.44	3.05	5.84	4.4E − 10
***Polygenic Scores`***					***p*_BH**
SCZ	49,873	− 0.58	− 0.81	− 0.34	5.9E − 05
CAD	49,873	0.49	0.25	0.72	8.4E − 04
CVD	49,873	0.44	0.20	0.67	3.4E − 03
***Genotype***					***p***
rs4149056 TT	36,079	*ref*	-	-	-
rs4149056 TC	12,759	0.09	− 0.45	0.62	7.56E − 01
rs4149056 CC	1162	− 0.002	− 1.56	1.55	9.98E − 01

Positive estimates (Coef: Coefficient) have increased PDC

^ Age at first statin: age at the first prescription by GP

^*^Multimorbidity includes 84 diseases including cardiovascular diseases. No add. Disease represents patients with no disease prior to their first statin prescription

`Three of 36 polygenic scores derived by Genomics *PLC,* tested are significant after The Benjamini–Hochberg multiple testing adjustments. The units are scaled; distribution has a zero mean and unit variance. *SCZ,* Schizophrenia, *CAD,* Coronary Arteria Disease, *CVD*, Cardiovascular Disease. Polygenic scores are continuous, and genotype are tested in European-like participants only to minimize population stratification effect

### GWAS

We performed GWAS analysis of two PDC traits, using REGENIE to analyse 16 million genetic variants with MAF > 0.001 and INFO > 0.3. One genetic variant was significantly (*p* < 5*10^−8^) associated with “PDC until LDL1” in linear regression models: rs548267220 (C > G, MAF = 0.006, INFO = 0.8, BETA = 12.7, *p* = 4.8*10^−8^), intronic for gene *RUNX3*. Two genetic variants were significantly (*p* < 5*10^−8^) associated with “PDC > 95% vs. ≤ 95%” in logistic regression models: rs9439705 (G > A, MAF = 0.92, INFO = 0.99, BETA = − 0.13, *p* = 1.4*10^−8^), located 30 kbp from *KLHDC7A*, and rs75103961 (C > T, MAF = 0.002, INFO = 0.92, BETA = − 0.77, *p* = 4.0*10^−8^), intergenic on chromosome 12. For details see Additional file 2: Table 12.

rs548267220 has not appeared in the GWAS catalog previously (5th Nov 2024), though there are 142 entries for *RUNX3* including haematological traits, kidney function, allergic diseases, blood pressure, and obesity. Neither rs9439705 nor rs75103961 have appeared in the GWAS catalog previously (5th Nov 2024). *KLHDC7A*, encoding protein Kelch domain containing 7 A, has previously been linked to LDL cholesterol, kidney function, and other traits in the GWAS catalog.

Genetic variants (e.g. rs79860430, rs77855582, rs73089338, rs504365, rs2247256) associated with muscle symptoms previously [36] did not reach statistical significance (nominal *p* > 0.05) in our GWAS analysis of binary PDC in year1 (≤ 95%, > 95%) (Additional file 2: Table 13).

### Time-to-event analysis

We analysed up to 55,784 patients across three PDC groups (low: < 50%, moderate: 50–95%, high: > 95%). Hypertension was the most prevalent disease (43%). Key differences between PDC groups included CHD (19% in low PDC, 17% in high PDC), cataract (8% in low PDC, 10% in high PDC), and chronic kidney disease (8.5% in low PDC, 10% in high PDC) (Additional file 2: Table 14).

Patients with high PDC had a decreased risk of ischemic stroke (HR 0.67, 95% CI 0.54–0.83, *p* = 10^−2^), coronary heart disease (HR 0.78, 95% CI 0.73–0.84, *p* = 2 × 10^−8^), transient ischemic attack (HR 0.79, 95% CI 0.68–0.92, *p* = 4 × 10^−2^); but an increased risk of cataract (HR 1.20, 95% CI 1.07–1.34, *p* = 4 × 10^−2^), iron deficiency anaemia (HR 1.3, 95% CI 1.09–1.54, *p* = 5 × 10^−2^; women: HR 1.32, 95% CI 1.02–1.72, *p* = 0.03; men: HR 1.26, 95% CI 1.01–1.58, *p* = 0.04), chronic kidney disease (HR 1,19, 95% CI 1.06–1.32, *p* = 4 × 10^−2^) and enthesopathy of the upper limbs (HR 1.16, 95% CI 1.06–1.26, *p* = 4 × 10^−2^) compared to those with low PDC (Table 3). *P*-values were adjusted using the Benjamini–Hochberg correction method.

**Table 3:** Associations between future diseases and PDC (low/high) PDC > 95 versus PDC < 50

Outcome	% high PDC _(low PDC)_	*n* case _high PDC_	*n* case _total_	HR	95%	CI	p_bh
CHD	16.62 (18.56)	5455	9421	0.78	0.73	0.84	2.E − 08
Isch Stroke	1.56 (2.30)	513	928	0.67	0.54	0.83	1.E − 02
Cataract	9.68 (7.81)	3177	5237	1.20	1.07	1.34	4.E − 02
CKD	10.04 (8.57)	3297	5370	1.19	1.06	1.32	4.E − 02
Enthesopathy U	12.62 (12.66)	4144	7162	1.16	1.06	1.26	4.E − 02
TIA	4.00 (4.73)	745	2221	0.79	0.68	0.92	4.E − 02
IDA	3.93 (3.38)	1289	2145	1.30	1.09	1.54	5.E − 02

We performed a time-to-event analysis in statin patients, starting 3 months post-initiation. Patients exited the model at either the event date or the end of follow-up. The model adjusted for sex, age at first statin use, education, assessment centre, and prevalent diagnosis of the event (binary). We compared adherence levels (≤ 50%, 50–95%, and > 95%), showing here on results for the high adherence group (PDC > 95%)

Outcomes were derived from General Practice (GP) and hospital records

Abbreviations: *CHD* coronary heart disease, *Isch* ischemic, *CKD* chronic kidney disease, Enthesopathy *U* upper, *TIA* transient ischemic attack, *IDA* iron deficiency anaemia, % prevalence, *n* total number of cases, *HR* hazard ratio, p_bh Benjamini–Hochberg-corrected *p*-value

## Discussion

Using real-world data, we identify substantial variability in the time to first on-treatment LDL-c and adherence to statin medication, highlighting a gap between NHS guidelines and the actual level of achieved treatment response monitoring. Our work highlights important predictors of reduced statin effectiveness, such as pharmacogenetic variants, polygenic scores, but most of all, sustained adherence to statin medications. Higher adherence to treatment was associated with greater adherence to therapy overall, leading to more significant LDL-c reductions over time. This suggests that improved service provision and patients’ factors—such as increased statin use, better understanding of treatment by patients, more frequent GP visits for LDL-c checks, and tailored treatments addressing low-adherence risk factors—could enhance efficacy, safety, and long-term patient outcomes.

When quantifying adherence using the proportion of days covered (PDC) measure, we assumed that patients adhered to their prescribed medication regimens. While PDC typically refers to the proportion of days a patient is covered by medication and is capped at 100% [37], our calculation allowed values above 100% to account for scenarios such as multiple-tablet daily regimens. However, since adherence was evaluated using a binary threshold (≤ 95% vs. > 95%), this methodological distinction is unlikely to meaningfully affect our results. However, the UKB lacks detailed data on actual medication intake and GP dosage instructions (e.g. whether a patient is taking 2 × 40 mg versus 1 × 80 mg). Therefore, we restricted our PDC analysis to patients with a PDC of less than 200%, under the assumption that they might be taking two tablets per day as prescribed. As a result, our adherence rate is higher than in previous Finnish cohorts, likely due to their smaller PDC cut-off and the fact that 96% of statin users in Finland were prescribed one tablet daily [38, 39]. Nevertheless, PDC is widely used and regarded as the gold standard for measuring medication adherence in observational studies, particularly for long-term treatments like lipid-lowering drugs [37, 40]. In the UK healthcare system, prescriptions are typically collected from a pharmacy shortly after issue. Thus, monthly GP prescribing patterns may serve as a reasonable proxy for medication availability (if someone is receiving regular prescriptions (e.g. monthly) and those are recorded in the electronic health record, it strongly suggests they are likely collecting the medication [41]—especially since getting a prescription usually requires action from the patient (request or visit). While PDC may not fully capture adherence behaviour at the patient level, the extent to which LDL-c change is strongly predicted by our PDC measure indicates that it is capturing the underlying relationship between medication adherence and effectiveness of treatment. Different PDC cut-offs have been used previously, we chose the > 95% PDC threshold because it splits the population into two groups with distinct LDL-c dynamics: leading to a 40% reduction in primary prevention and an average 2 mmol/L reduction in secondary prevention, aligning with NICE guidelines and enhancing clinical relevance.

To add further weight to this, our finding of an increased risk of iron deficiency anaemia (IDA) in patients with higher statin adherence (HR 1.30: 95% CI 1.12–1.70) is consistent with previous research: a hypothesis-free analysis of a Korean cohort (7,847 statin users vs. 39,235 non-users) similarly identified a higher risk of IDA (95% CI 2.11–12.03) linked to statin use among over 100 tested diseases, supported by Mendelian Randomization analysis [42]. The possible mechanism between iron and statins can be linked to statin’s capacity to favourably modulate iron homeostasis [43], which might be beneficial for CVD risk as abnormal iron accumulation in cells increases oxidative stress, vascular inflammation, and the development of atherosclerosis [43, 44]. Additionally, we observed the increased risk in both sexes, suggesting it is not solely driven by the higher baseline risk of IDA in women.

A review of over 313,200 patients [45] found a moderate increase in cataract risk with statin use in cohort studies, which had long follow-up periods (~ > 5 years) and various baseline characteristics, while case–control studies and randomized trials showed no significant risk. Although our findings support previous cohort studies with long-term follow-up, the review concludes that there is likely no significant link between statin treatment and cataracts, emphasizing that the benefits of statin therapy outweigh potential risks.

In exploratory analysis to evaluate the effect of a sustained PDC intervention on LDL-c reduction using longitudinal causal models, we made a critical assumption that all confounders between PDC and LDL-c across three time points were observable and could be controlled for in the analysis using inverse probability of treatment weighting. The resulting estimates suggest that approximately 35% reductions in LDL-c are eminently achievable in the UKB population under full adherence. Ensuring high adherence in the most recent period was estimated to have, unsurprisingly, the largest influence on patients’ LDL-c reduction, but ensuring high adherence in their initial treatment phase was interestingly the next most effective way to achieve this. This again highlights the importance of early follow-up monitoring after statin initiation. A possible direction for future research is to utilize pharmacogenetic predictors of treatment adherence within a Mendelian randomization framework [46, 47], to facilitate this analysis under a relaxation of the observable confounder assumption. Unfortunately, we did not identify any suitably strong genetic predictors of PDC in the UKB primary data reaching genome-wide significance. This is consistent with findings from the FinnGen/EstoniaBiobank study [20], which also failed to identify any genome-wide significant genetic variants associated with PDC in statin users [20]. Future work will expand this search to include other cohort studies and to consider other measures of adherence beyond PDC.

A PDC below 50% is particularly important, as it indicates that even if patients are taking 1/2 tablets daily, their adherence is still insufficient to fully meet the prescribed regimen. Consequently, patients with low adherence had a 27% higher risk of coronary heart disease and a 50% increased risk of ischemic stroke compared to those with high adherence. Understanding and improving adherence is crucial to reducing cardiovascular events. Predictors of low adherence to statins included genetic liability to schizophrenia. Likewise, a study from the Estonian Bank [48] highlighted that genetic liability to schizophrenia was associated with schizophrenia diagnoses in this cohort. For these patients, higher adherence to antipsychotic medications reduced metabolic syndromes, which are known to elevate the risk of heart disease and stroke. Our research suggests that this could be due indirectly to higher levels of statin adherence rather than a direct effect of antipsychotic medication. A logical explanation for the link between genetic liability to schizophrenia and low PDC is the increased burden of taking medication on time for those with mental health conditions. We suspect that the nominal association between PDC and the Alzheimer’s disease polygenic score may be largely driven by pleiotropic effects of APOE-ε4, which is a major genetic risk factor for both Alzheimer's disease and altered lipid metabolism.

We limited the genetic analysis to individuals of European ancestry, which may limit generalisability to other populations but reduces stratification bias. When investigating genetic predictors of on-statin LDL-c response, we initially focused on the pharmacogenetic polygenic risk score reported by Mayerhofer et al. [49]. We did not find the score to be predictive of an LDL-c reduction as a whole. rs2900478, a single SNP included in the polygenic score, was highly correlated (*r*^2^ = 0.96) with the well-known statin pharmacogenetic variant SLCO1B1*5 (rs4149056). A previous GWAS of statin-associated muscle pain by Carr et al. [36] identified SLCO1B1*5 associated with muscle symptoms, a potential adverse event for non-adherence [36]. Consistent with our previous work, where we found that SLCO1B1*5 was associated with discontinuation of statin therapy [19], we here report that *5 is associated with attenuated LDL-c reduction following statin initiation. We found no association between SLCO1B1*5 and PDC (adherence) perhaps because, by definition, those with multiple prescriptions meeting inclusion criteria have not yet discontinued. When taken together, our analyses support the known pharmacogenetic effect of *5 on statin effectiveness, and potential impact on adverse events.

Other variants reported as nominally associated with muscle symptoms by Carr et al. were not associated with PDC in our analysis. The LPA variant rs10455872, previously associated with LDL-c response [27] and included in the polygenic score by Mayerhofer et al., was linked to reduced LDL-c reduction in our analysis, but also to higher baseline LDL-c, suggesting its effect may reflect general LDL-c levels rather than a statin-specific response.

## Conclusions

This is the first study, to our knowledge, that uses a large cohort with linked genetics and primary care health records to examine the effect of statin adherence on LDL-c change and long-term outcomes, and identify risk factors for adherence using genetic and causal inference models. Our work emphasizes insufficient LDL-c control with a need for LDL-c monitoring in line with clinical guidelines, and a clear clinical benefit of adhering to prescribing guidance, which reinforces the importance of patient education on statin indication and adherence. It also emphasizes the clear clinical benefits of adhering to prescribed statin therapy, not only in terms of LDL-c reduction but also in reducing the downstream risks of related diseases, with high adherence being associated with a decrease in the cerebro/cardio-vascular diseases.

## Supplementary Information

Additional file 1: Table 1- Dose intensity classification based on the NICE Guideline. Table 2-Prevalence of the dose intensity groups in individuals prescribed statins in the UK Biobank GP data. Fig. S1-Associations between time to first GP LDL measure following statin initiation and LDL change. Fig. S2-Associations between time to first GP LDL measure following statin initiation and LDL change by PDC groups. Fig. S3-Quadratic model of LDL reduction estimated by SLCO1B1 rs4149046 genotype. Fig. S4-Quadratic model of LDL reduction estimated by LDL-response variants.

Additional file 2: Table 1-ICD10 codes for the cardiovascular diseases. Table 2-Details of the polygenic scores ascertained from UK Biobank release and systematic evaluation of optimized polygenic risk scores for 53 diseases and quantitative traits. Table 3-Read codes for low-density lipoprotein in UK Biobank primary care data. Table 4-Polygenic Scores and LDL change associations (LDL1/LDL0) in statin patients. Table 5-The weighted Pearson correlations between the exposure and confounding covariates with the CBPS weights. Table 6-The weighted Spearman correlations between the exposure and confounding covariates with the CBPS weights. Table 7-The weighted OLS regression results with the CBPS weights. Table 8-The weighted OLS regression results with the npCBPS weights. Table 9-Sensitivity analysis: the weighted OLS regression results with the CBPS weights in the primary prevention subgroup. Table 10-Sensitivity analysis: the weighted OLS regression results with the npCBPS weights in the primary prevention subgroup. Table 11-Polygenic scores of traits and diseases associations with PDC in statin patients. Table 12-GWAS results for two PDC phenotype. Table 13-GWAS Results for PDC Binary Phenotype (< = 95% vs > 95%) and Previously Reported Nominal Associations with Muscle Symptoms. Table 14-Time to event analysis in statin patients for incident clinical outcomes.

## Data Availability

No datasets were generated or analysed during the current study.

## Abbreviations

AHA: American Heart Association
BNF: British National Formulary
CBPS: Covariate Balancing Propensity Score
CVDs: Cardiovascular diseases
DM + D: Dictionary of Medicines and Devices
GP: General Practitioner
IDA: Iron deficiency anaemia
IPTW: Inverse probability of treatment weighting
LDL-c: Low-density lipoprotein cholesterol
MAF: Minor allele frequencies
NICE: The UK National Institute for Health and Care Excellence
npCBPS: Non-parametric Covariate Balancing Propensity Score
PDC: Proportion of days covered
PGS: Polygenic scores
RCT: Randomized controlled trials
