# Supplementary material for: Understanding the causes and consequences of low statin adherence: evidence from UK Biobank primary care data
Source: BMC Med. 2025 Jul 22;23:436. doi: 10.1186/s12916-025-04228-2 (PMC12285031; doi:10.1186/s12916-025-04228-2)

**Additional File 1**

## Methods

### Cross sectional modelling of LDL reduction explained by week of follow up

We tested associations between LDL change and the time interval between their pre- and post-treatment measurements, using a linear regression model, adjusted additionally for dose group of first atorvastatin and simvastatin prescribed in year 1. Specifically:

log(Y) = $\beta_{0}+ \beta_{1}.factor\left( T \right)$

Y = LDL change (LDL1/LDL0)

T = Time interval between their pre- and post-treatment measurements

We organised doses into a categorical variable based on NICE guidelines (23) for dose equivalence, as shown below:

**Table 1- Dose intensity classification based on the NICE Guideline**

| **Drug** | **10 mg** | **20 mg** | **40 mg** | **80 mg** |
| --- | --- | --- | --- | --- |
| Simvastatin | low | middle | Middle | High |
| Atorvastatin | middle | high | High | High |

**Table 2- Prevalence of the dose intensity groups in individuals prescribed statins in the UK Biobank GP data**

| **Dose intensity** | **N total** | **%** | **N (in both LDL0 and LDL1 available)** | **%** |
| --- | --- | --- | --- | --- |
| low | 5,808 | 7.7 | 2,355 | 6.4 |
| middle | 57,293 | 76.3 | 29,560 | 80.4 |
| high | 12,013 | 16.0 | 4,833 | 13.2 |
| total: | 75114 |  | 36,748 |  |

We repeated this analysis in ${PDC}_{1}$ groups (<=95% and >95%).

### Modelling LDL reduction by genotype, PDC and time of follow up measure.

We tested associations between LDL change and rs4149056 using a linear model that allowed for a quadratic effect of time to follow up on treatment in European-like participants and an interaction between time on treatment and rs4149056:

E[$Y]=\beta_{0}+ \beta_{1}.T_{off}+ \beta_{2}.T_{on}+ \beta_{3}.{(T_{on})}^{2}+ \beta_{4}. G+ \beta_{5}. T_{on}G+ \beta_{6}.{(T_{on}.G)}^{2}$ (1)

$Y$ *= LDL change* (LDL1/LDL0)

$T_{off}$ *= Time between pre-statin LDL measurement and the statin initiation*

$T_{on}$ *= Time to first LDL measures after the statin initiation x (dose group).*

$G$ *= rs4149056 (number of copies)*

Model (1) additionally adjusted for 10 genetic principal components (PCs).

Next, we tested the association between LDL change and ${PDC}_{1}$ using model (1), but with PDC in place of the G variable, rs4149056. This analysis did not adjust for genetic PCs but accounted for the prevalence of CVD before statin use, including abdominal aortic aneurysm, atrial fibrillation, coronary heart disease, peripheral arterial disease, all types of stroke, and thromboembolic disease.

### Causal modelling of hypothetical LDL reduction via sustained PDC intervention

Let the vector $\bar{PDC}_{ik}$ denote the adherence history of patient $i$ up to time point $k$ so that: $\bar{PDC}_{ik}=({PDC}_{i1}, \ldots, {PDC}_{ik})$. We define ${LR}_{ik}^{\boldsymbol{a}}$ as the potential LDL reduction at time point $k$ given that $\bar{PDC}_{ik}$ takes the value $\boldsymbol{a}$**.** We assume that ${PDC}_{i1}$ to ${PDC}_{ik}$ exert linear additive effects on ${LR}_{ik}$. More concretely, for each time point k, we define the estimand of interest as the contrast between the potential outcomes under PDC history profile $\boldsymbol{a.+1}$ and $\boldsymbol{a}$**.**, which we assume obeys the following parametric relationship:$E\left( {LR}_{ik}^{\boldsymbol{a+1}} \right)\boldsymbol{-}E\left( {LR}_{ik}^{\boldsymbol{a}} \right)\boldsymbol{=}\sum_{j=1}^{k} \beta_{k}\boldsymbol{(}j\boldsymbol{)}$

where $k=1, 2, 3$ and $\beta_{k}\boldsymbol{(}j\boldsymbol{)}$ represents the causal effect of ${PDC}_{ij}$ on ${LR}_{ik}$ with $j=1, \ldots, k$. We aim to estimate $\beta_{k}\boldsymbol{(}j\boldsymbol{)}$ with the inverse probability of treatment weighting (IPTW) method under the assumption that all the confounders between PDC and LR are observable and can be controlled for in the analysis. Since we have a continuous exposure variable PDC, according to (20) and (30) the weight for individual $i$ at time point $k$ is given by

$$W_{i,k}=\prod_{j=1}^{k} w_{i,j}= \prod_{j=1}^{k} \frac{f({PDC}_{i, j})}{f\left( {PDC}_{i, j} \right| L_{i, j})}$$

with $k=1, 2, 3. {f(PDC}_{i, j})$ and $f\left( {PDC}_{i, k} \right| L_{i, j})$ are the marginal and conditional density functions respectively. ${PDC}_{i, j}$ is the observed value of the PDC for individual $i$ at time point $j$ and $L_{i, j}$ summarizes all fixed covariates mentioned in the main text, the previous exposure ${PDC}_{i, j-1}$ and previous outcome ${LR}_{i, j-1}$ (for $j=2, 3$), and the patient visit time, denoted by$t_{i,j}$.

We estimate each $w_{i,j}$ ($j=1, 2, 3$) with the Covariate Balancing Propensity Score (CBPS) method proposed by (30) The method was implemented with the R functions ‘*CBPS’* and ‘*npCBPS’* (for the non-parametric option) from the R package ‘*CBPS’ (https://cran.r-project.org/web/packages/CBPS/index.html).* We ran the functions with default settings except that we set ‘method = exact’ for CBPS. For each $j=1, 2, 3$, we estimated $w_{i,j}$ by fitting the model of ${PDC}_{i, j} \sim L_{i, j}$. For a given time point $k$ ($k=1, 2, 3$), $W_{i,k}$ is then obtained with $W_{i,k}=\prod_{j=1}^{k} w_{i,j}$. We multiplied each $W_{i,k}$ by ${10}^{13}$ for the second stage weighted OLS to avoid zero truncation due to machine tolerance issues. In the second-stage regression, we omit the intercept.

## Results

**Figure S1 –** **Associations between** **time to first GP LDL measure following statin initiation and LDL change**


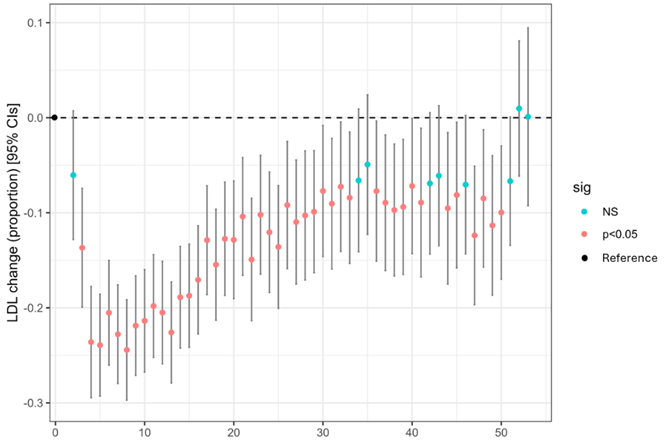


Weeks following statin initiation

This plot illustrates associations between LDL reduction and visit days, based on available LDL-C data for each week, not individual-level data. The maximum reduction occurs between weeks 5-8 of 26% (95%CI 23-29).

**Figure S2** - **Associations between** **time to first GP LDL measure following statin initiation and LDL change by PDC groups**

**>95% PDC <=95% PDC**

**
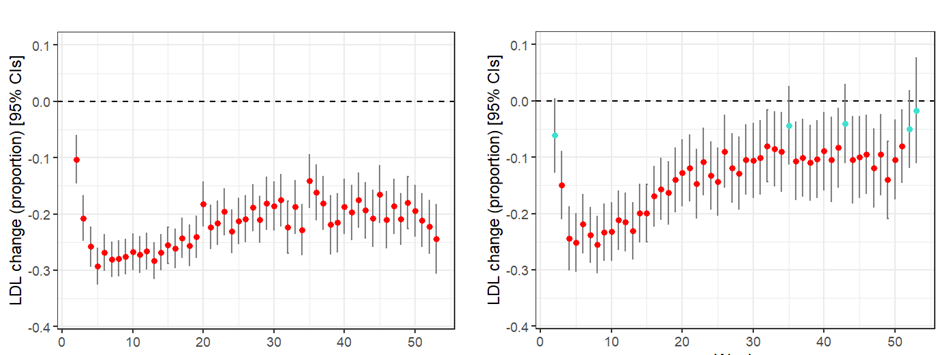
**

Weeks until the first post-statin LDL measurement in the GP clinic

This plot illustrates the associations between LDL reduction and GP visit days, based on available LDL-C measurement post stain initiation for each week in two PDC groups.

**Figure S3 - Quadratic model of LDL reduction estimated by SLCO1B1 rs4149046 genotype**


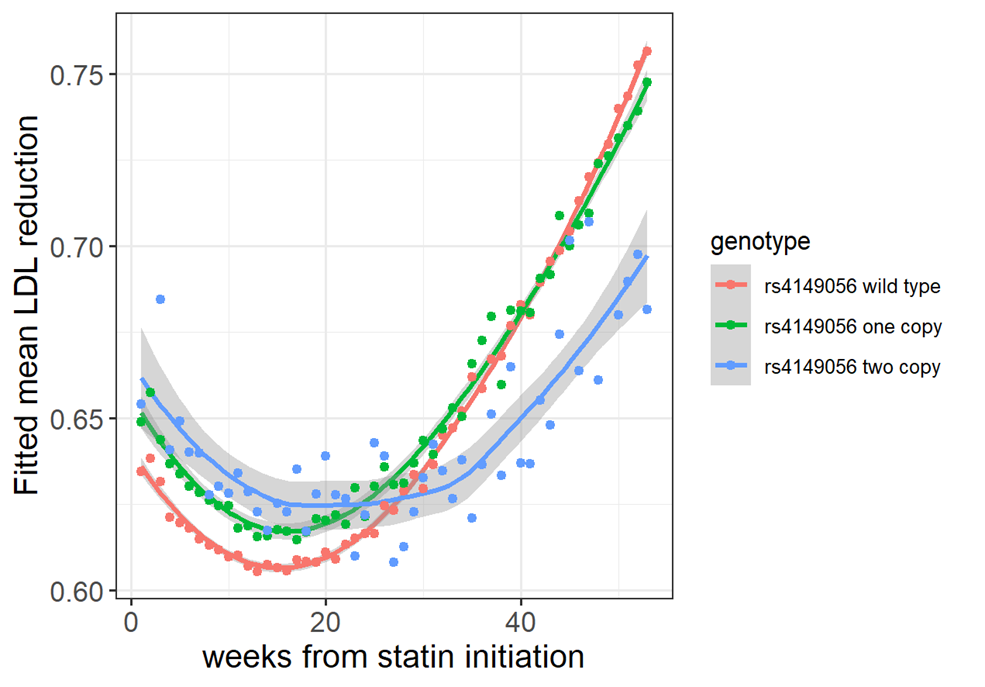


The quadratic model revealed that those with the rs4149046 CC allele had the smallest LDL-c reduction in the in year 1 (mean maximum reduction around 37%) versus TT allele (mean maximum reduction around 39.5%).

**Figure S4 - Quadratic model of LDL reduction estimated by LDL-response variants**


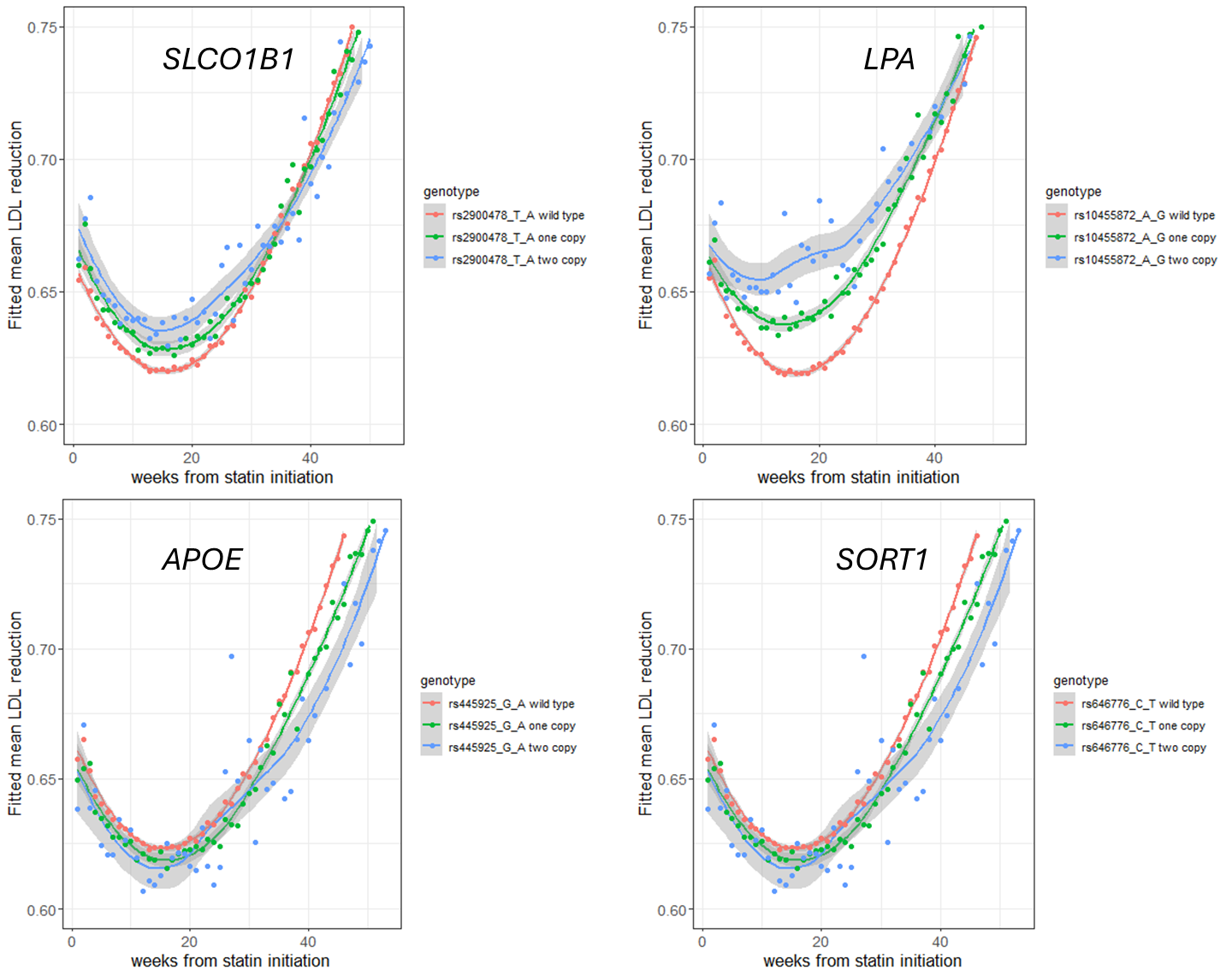

Supplement: Supplementary file 1 — Additional file 1: Table 1- Dose intensity classification based on the NICE Guideline. Table 2-Prevalence of the dose intensity groups in individuals prescribed statins in the UK Biobank GP data. Fig. S1-Associations between time to first GP LDL measure following statin initiation and LDL change. Fig. S2-Associations between time to first GP LDL measure following statin initiation and LDL change by PDC groups. Fig. S3-Quadratic model of LDL reduction estimated by SLCO1B1 rs4149046 genotype. Fig. S4-Quadratic model of LDL reduction estimated by LDL-response variants. [file 12916_2025_4228_MOESM1_ESM.docx]
